# Doping-Promoted Solar Water Oxidation on Hematite Photoanodes

**DOI:** 10.3390/molecules21070868

**Published:** 2016-07-01

**Authors:** Yuchao Zhang, Hongwei Ji, Wanhong Ma, Chuncheng Chen, Wenjing Song, Jincai Zhao

**Affiliations:** 1 Beijing National Laboratory for Molecular Sciences, Key Laboratory of Photochemistry, Institute of Chemistry, The Chinese Academy of Sciences, Beijing 100190, China; wxzyc@139.com (Y.Z.); hwji@iccas.ac.cn (H.J.); whma@iccas.ac.cn (W.M.); ccchen@iccas.ac.cn (C.C.); 2 University of Chinese Academy of Sciences, Beijing 100049, China

**Keywords:** water splitting, hematite, doping, charge transfer

## Abstract

As one of the most promising materials for solar water oxidation, hematite has attracted intense research interest for four decades. Despite their desirable optical band gap, stability and other attractive features, there are great challenges for the implementation of hematite-based photoelectrochemical cells. In particular, the extremely low electron mobility leads to severe energy loss by electron hole recombination. Elemental doping, i.e., replacing lattice iron with foreign atoms, has been shown to be a practical solution. Here we review the significant progresses in metal and non-metal element doping-promoted hematite solar water oxidation, focusing on the role of dopants in adjusting carrier density, charge collection efficiency and surface water oxidation kinetics. The advantages and salient features of the different doping categories are compared and discussed.

## 1. Introduction

Water oxidation has been considered as the Holy Grail of chemistry in the 21th century, as it is the efficiency-controlling step of light-driven overall water splitting (2H_2_O → 2H_2_ + O_2_) to store solar energy in chemical bonds [1,2,3,4,5,6]. Photoelectrcochemical (PEC) cells are a promising technology to realize solar water splitting. In the PEC configuration, water oxidation occurs on the photoanode [2,3,6,7,8,9,10]. Metal oxide semiconductors such as TiO_2_ [11,12], BiVO_4_ [13], WO_3_ [14], and α-Fe_2_O_3_ (hematite) are the most widely explored materials as suitable photoanodes [2,3,6,7,9,10,15,16,17,18,19,20,21,22,23,24]. Hematite is promising due to its abundance, low cost, stability and visible light absorption [2,3,6,9,25,26,27,28,29,30]. Ideally, anodic photocurrent onset is close to its flatband potential (0.3–0.4 V vs. RHE) and the photocurrent density approaches a plateau of 12.6 mA/cm^2^ (calculated by hematite absorption and AM 1.5 G solar spectrum) [2,31]. However, the ultrashort excited states (carrier) lifetimes [15,32], low electron mobility [33] and a hole diffusion length of 2–4 nm [34] impair charge collection at the surface; the mid-bandgap states pinned Fermi level [18,35], as well as the sluggish interfacial hole transfer kinetics require a large overpotential to drive water oxidation [2,15]. Many strategies tackling these limitations have been developed, including morphology/nanostructure control to decouple the light penetration depth and hole diffusion length, and elemental doping to facilitate carrier transport. The keys are efficient electron hole separation, hole collection at the surface and rapid hole transfer at hematite/electrolyte interface for catalytic water oxidation [2,3,6,9]. Significant progress has been made in achieving higher water oxidation activity and solar to hydrogen (STH) efficiency. To date, the champion hematite-based PEC cell gives a photocurrent density of 4.68 mA/cm^2^ at 1.23 V vs. RHE [36].

One of major obstacles for efficient solar water oxidation is the extremely low electron mobility of hematite (10^−2^ cm^2^·V^−1^·s^−1^) [2]. According to DFT calculations, the conduction band edge of hematite is highly localized on the Fe 3d orbital, leading to an extremely heavy electron effective mass [37,38]. DFT calculation also predicts that the incorporation of heteroatoms could reduce the electron effective mass and improve the electron mobility [37,38]. Detailed fundamentals of the dopant- induced property change will be addressed hereinafter. Dopants are also found to affect other properties of hematite (crystalline disorder, morphology, etc.) or act as catalytic sites, playing multiple roles in boosting PEC water oxidation efficiency.

Here we review selected researches with major contributions to the doping-promoted water oxidation on hematite photoanodes, under the categories of metal and non-metal dopants [2,3,6,9]. For metal element doping, *n*-type dopants are represented by Ti^4+^ [39,40,41,42,43,44,45], Sn^4+^ [46,47,48,49,50,51], Zr^4+^ [52,53] and Pt^4+^ [54,55,56]; *p*-type dopants include Mg^2+^ [57,58], Zn^2+^ [59,60,61,62,63], Ag^+^ [64], Cu^2+^ [65]; and there is also nonisovalent substitutional doping by Al^3+^ [66]. Studies on non-metal element doping are dominated by Si [31,67,68,69,70,71] and P [33,34], which give superior activity. Indeed, theoretical calculations have suggested that non-metal element doping is a better choice to improve electron transport [31,33,67,68,69,70]. The role of heteroatoms in the hematite host will be elucidated by detailed analysis of carrier density and charge transfer kinetics, in the bulk and at hematite/electrolyte interface. Alternative mechanisms for efficiency enhancement are also discussed.

## 2. Metal Element Doping

### 2.1. n-Type Doping

**Figure 1:**
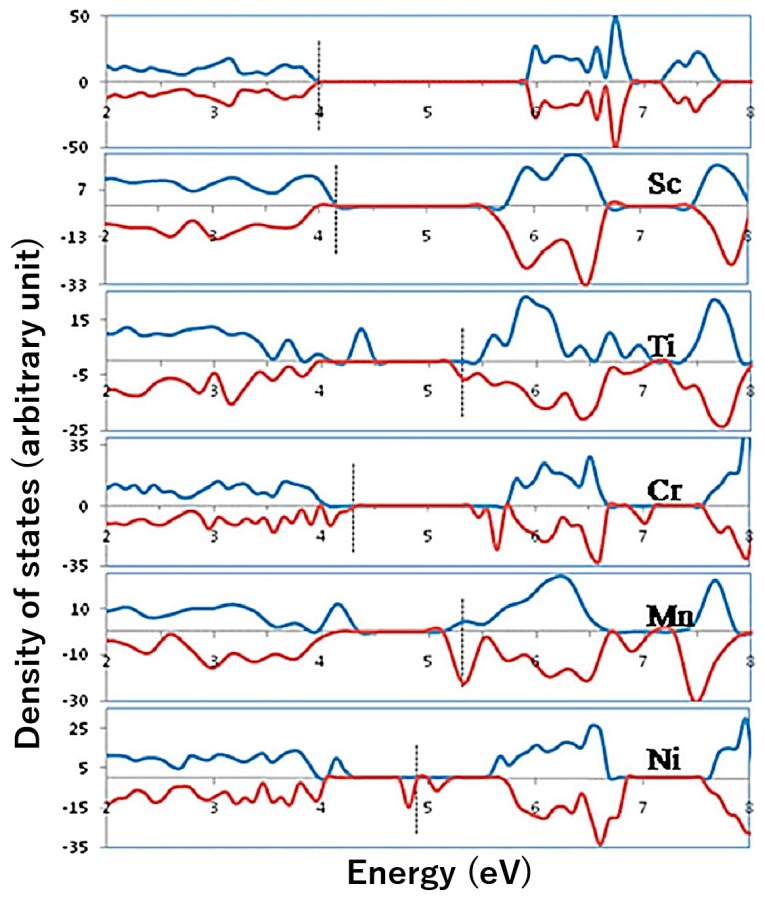
Total electronic DOS plot for pure Fe_2_O_3_ and Fe_2_O_3_ incorporated with Sc, Ti, Cr, Mn, and Ni at substitutional sites. The states are aligned with respect to the O 1s core level and the highest occupied state is indicated by the dashed vertical line in each case. Adapted with permission from [37]. Copyright 2010 American Institute of Physics Publishing.

Ti-doping is the most widely adapted approach to improve the water oxidation efficiency [39,40,41,42,43,44]. A survey of 3d transition metal (Sc, Ti, Cr, Mn, Ni) doping by DFT calculation revealed that incorporation of Ti gives the most dispersive conduction band density of state (Figure 1). In contrast to pristine hematite, doping introduces titanium d and s orbital character in the conduction band minimum (CBM), which reduces the effective mass of electron and enhance the electron mobility. The lattice strain (shorter intraplane Fe-Fe distance) in Ti-doped hematite further increases the polaron hopping probability [37]. These electronic features provide mechanistic fundamentals for the doping-facilitated charge transfer as well as the improved STH efficiency. Cr^3+^ doping shows a similar effect, however much less work has been done on Cr-doped hematite [72,73], which may be a promising endeavor in the future.

**Figure 2:**
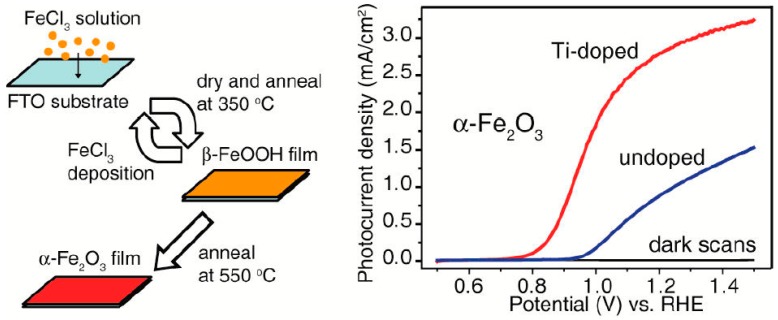
Synthesis process of deposition-annealing (**left**) and the J-V scan of the undoped and Ti-doped hematite photoaondes (**right**). Adapted with permission from [39]. Copyright 2011 American Chemical Society.

Several reports on Ti-doped hematite have demonstrated high photocurrent density in PEC water oxidation under AM 1.5 G simulated sunlight irradiation (the standard light source unless otherwise noted) [39,40,41,42,43,44,45]. In 2011, Wang et al. reported a novel deposition-annealing (DA) procedure to prepare Ti-doped hematite nanoparticles on fluorine-doped tin oxide (FTO) substrates, with titanium butoxide as the Ti precursor. At the surface of Ti-doped samples, Ti atoms are bonded to oxygen, with a binding energy consistent with that in TiO_2_ as suggested by X-ray photoelectron spectroscopy (XPS). The optimized photoanode (10% Ti atom percentage) achieved a photocurrent density of 2.8 mA/cm^2^ at 1.23 V vs. RHE (Figure 2). Notably, for Ti-doped photoanodes, the photocurrent onset potential was shifted cathodically by ~100–200 mV compared to the undoped hematite. The authors attributed the high activity to a two order of magnitude increase in carrier density and the reduced electron-hole recombination, based on Mott-Schottky and picosecond transient absorption measurements [39]. The *J-V* profile could be rationalized by the difference in bias- dependent electron-hole recombination of pristine and Ti-doped photoanode [23]. The cathodic shift of onset potential was ascribed to the surface TiO_2_-like motifs with O_2p_ holes (compared to the Fe^3+^ character of valence band holes) that enhanced hole transfer kinetics for water oxidation [2,15,74]. That is, Ti doping is able to lower the overpotential by forming TiO_2_ heterostructures on the hematite surface.

Deng et al. developed a facile hydrothermal method to synthesize Ti-doped hematite on FTO with TiCN as the Ti precursor [45]. The doped photoanode showed favorable sea urchin-like nanostructures, whereas pure hematite nanorods were vertically aligned on FTO substrates. At 1.23 V vs. RHE, the photocurrent density reached 1.91 mA/cm^2^, or 2.5 times higher than the pristine hematite. The higher water oxidation activity was again attributed to the doping-enhanced carrier density, as evidenced by a decrease in the unoccupied states of the conduction band from X-ray absorption spectroscopy (XAS). It is interesting to note that in the Ti-doped hematite, the Ti 2p_3/2_ binding energy deviated by ~0.7 eV from that of TiO_2_, which is quite different from the above-mentioned Ti-doped nanoparticles and may account for the less pronounced change in photocurrent onset potential.

To establish the correlation between the effects of the incorporated Ti on the electronic structures of Ti-doped hematite, Kronawitter et al. performed synchrotron-based soft X-ray absorption measurements on five types of Ti-doped samples fabricated by different methods [39,40,41,42,43,44,45]. All samples demonstrated spectral characters that indicate mixing of Ti s and d states in the conduction band, as predicted by DFT calculations [37]. The degree of orbital mixing is found to be more pronounced in the near-surface region [43], consistent with the higher doping levels near the surface according to the XPS depth profile result [41]. However, in contradiction to the theoretical calculation [38], conversion from Fe^3+^ to Fe^2+^ was not observed by Fe L_2,3_-edge absorption spectra in any case. Although the extra electrons donated by heteroatoms are proposed as one dominant mechanism for doping to improve the electron mobility and charge collection, variation in the iron valence state has rarely been observed experimentally for metal-doped hematite [39,43]. However, conversion of Fe^3+^ to Fe^2+^ was evidenced in the P-doped hematite developed by our group (see Section 3.2 for details).

**Figure 3:**
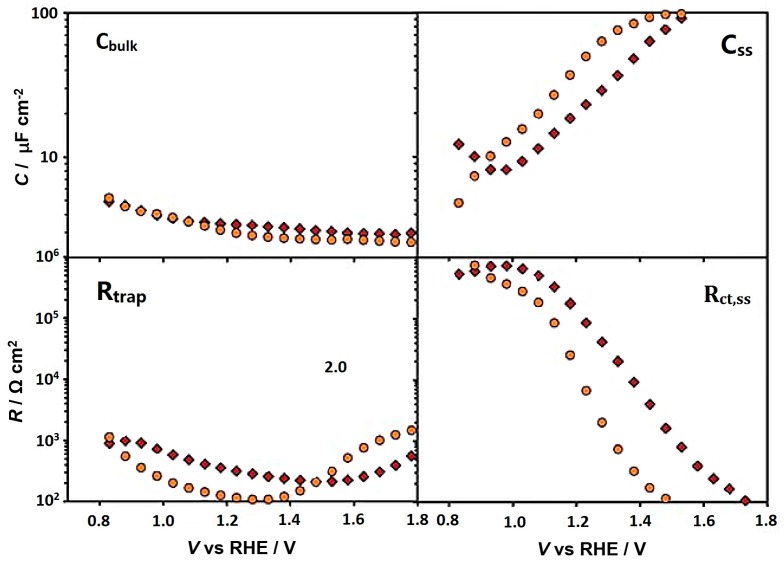
Impedance spectroscopy parameters calculated from fitting the EIS data to the equivalent circuit for an undoped (red diamonds) and a doped (orange circles) 300 cycle electrode. Adapted with permission from [41]. Copyright 2013 Royal Society of Chemistry.

Although Ti-doping introduced increases in carrier density are usually related to the improved PEC water oxidation, other mechanisms may prevail. Zandi et al. synthesized Ti-doped hematite films with highly uniform planar morphology by atomic layer deposition (ALD) [41]. Doped photoanodes with a 3% Ti atomic percentage exhibited dramatic boosts in photocurrent density and more negative onset potential. By electrochemical impedance spectroscopy (EIS, Figure 3), the authors were able to quantify the enhanced hole collection efficiency in the bulk (low charge trapping resistance, R_trap_) and hole transfer/water oxidation kinetics (low interfacial charge transfer resistance, R_ct,ss_) at the surface. However, the carrier densities for undoped and Ti-doped hematite thin films were at the same level (4.0 × 10^18^–4.4 × 10^18^ cm^−3^). They attributed the improved hole collection efficiency to the resurrection of a dead layer (formed due to the lattice mismatch of FTO and hematite which causes a severe charge recombination) by the doped Ti atom. Ti doping was also hypothesized to give higher concentration of surface Fe^III^OH that is active in the subsequent water oxidation [41]. Another study on ultrathin hematite photoanodes by ALD demonstrated a similar effect of Ti to increase the hole collection efficiency [75]. A more significant band bending in the space charge layer was suggested to facilitate electron-hole separation.

These studies revealed that the Ti dopant improves charge transfer/collection efficiency by alternating both bulk and surface properties of hematite. Activity enhancement by surface change could originate from passivation that reduces electron-hole recombination at surface or faster surface hole transfer kinetics. In a Ti-doped hematite nanowires fabricate by post growth strategy with titanium *n*-butoxide precursor, it was found that Ti dopant passivates particular mid-band gap surface states and inhibits electron-hole recombination mediates by these states. Therefore, a higher efficiency of hole transfer to water was achieved. Intriguingly, Zr doping with the same treatment led to similar donor density, but the insignificant surface passivation gave much less photocurrent enhancement [51].

More recently, Monllor-Satoca et al. prepared Ti-doped hematite film by mixing of preformed hematite/titania nanoparticles followed by 700 °C calcination, gaining 15 times photocurrent enhancement at 10% Ti [76]. Characterizations by Mott-Schottky and EIS evinced increases in bulk donor density (N_d_) and surface states (N_ss_). Notably, N_ss_/N_d_ exhibited the same trend with %Ti as photocurrent and the relative importance of interfacial charge transfer (characterized by ratio of charge trapping resistance to surface charge transfer resistance). Such results clearly point to a favored surface charge transfer process owing to the Ti-introduced positive shift in surface state energy. Moreover, pseudobrookite (Fe_2_TiO_5_) forms near nanoparticle edges and the hematite/pseudobrookite heterojunction offer favorable cascades for vectorial diffusion of electrons and holes. This work highlights the multiple changes by doping and the interplay of these factors for optimized performance [76]. Fe_2_TiO_5_ is also a suitable n-type photoanode material [77]. It has a band gap of ~2.1 eV and the band energy levels allows formation of n-n junctions when combined with hematite. With such a configuration, Bassi et al. constructed a photoanode which gave photocurrent of 1.4 mA/cm^2^ (0.01 mA/cm^2^ for pristine hematite) [78]. Besides the band alignment facilitated bulk charge separation, surface states of Fe_2_TiO_5_ improved surface hole transfer kinetics was also proposed to boost the PEC water oxidation. The charge transfer dynamics in similar composite photoaonde was supported by independent transient absorption measurements [79].

**Figure 4:**
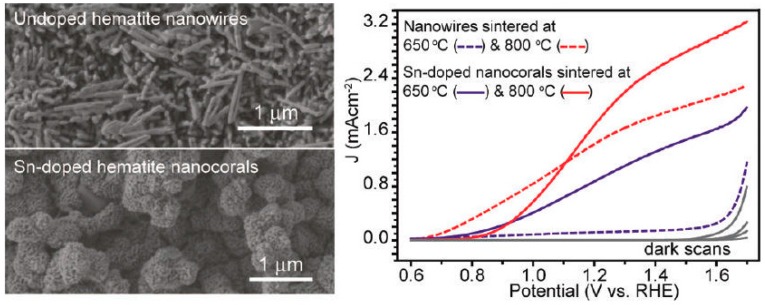
The morphology (**left**) and PEC activity (**right**) of the undoped and Sn-doped hematite photoanodes. Adapted with permission from [46]. Copyright 2012 American Chemical Society.

Sn-doping is another popular strategy to improve the activities of hematite photoanodes [46,47,48]. The effect of Sn was first realized in 2010, where the unintentional incorporation of Sn (via diffusion from FTO substrates to hematite) occurred at high annealing temperature (800 °C) [80]. This was re-visited systematically and confirmed by Ling et al. [46]. The Sn-doped hematite nanowires prepared by annealing at 800 °C show pronounced photocurrent density of 1.24 mA/cm^2^ at 1.23 V vs. RHE (Figure 4). The authors then developed a hydrothermal method to fabricate Sn-doped hematite nanocorals on FTO by adding tin(IV) chloride as the Sn precursor, further increasing the photocurrent density to 1.86 mA/cm^2^ at 1.23 V vs. RHE. It is important to note a 5-times higher photocurrent density for Sn-doped hematite sintered at 650 °C, compared to undoped photoanodes, which reinforces the critical role of Sn. The substantial enhancement in PEC efficiency was a synergistic effect of Sn-introduced carrier density, the increased light harvesting efficiency and the high surface area [46]. More thorough investigations on Sn-doping were performed on sputter-deposited hematite thin films. The highly uniform coverage allows for precise quantification of Sn content by secondary ion mass spectrometry (SIMS). Surprisingly, compared to low temperature treatment, high temperature annealing led to a one magnitude increase in Sn content, but a 10^3^ and 10^5^ increase in photocurrent density and conductivity, respectively. These results indicate that higher temperature treatment is crucial to activate Sn in hematite [47].

Frydrych et al. developed a facile spin-coating based method that allows fabricating photoactive Sn-doped hematite at lower annealing temperature (650 °C) [48]. A high tin loading level (20:100, Sn:Fe) was found to give over a 10-times increase in photocurrent. XPS and magnetization measurements were used to determine the doping level and effect of tin on the crystal structure. The low annealing temperature suggested an alternative dopant activation mechanism. Despite the increase in donor density, DFT calculations by Annamalai et al. indicated that Sn-doping creates localized shallow donor levels, which are detrimental for conductivity. Luckily, this could be overcome by sequential ex-situ Sn^4+^ and Be^2+^ doping. The co-doped hematite exhibits less localized CBM and smaller effective electron mass, leading to further improvement in charge transport and a 1.7 times enhancement in photocurrent comparing to Sn-Fe_2_O_3_ [51]. Zr was also reported to be a suitable co-dopant for Sn to achieve higher electrical conductivity and PEC water oxidation activities [81].

In addition to the beneficial effect on charge transport and hole trapping step, there are evidences for Sn dopant impacting the charge transfer at hematite/electrolyte interface. Dunn et al. developed a solution process route to fabricate Sn-doped photoanodes while maintaining the mesoporous worm-like morphology [49]. A much higher surface hole transfer efficiency in doped hematite was confirmed by photocurrent transient analysis. By intensity modulated photocurrent spectroscopy (IMPS), the authors identified that the increase in hole transfer rate, not surface passivation (suppression of electron-hole surface recombination), was responsible for the improved efficiency. Such a phenomenon was attributed to Sn-enrichment at the nanostructure surface, which may define the energetics of intermediates (surface states) involved in water oxidation. Later, Shinde et al. systematically investigated the properties of hematite in which Sn was incorporated via substrate diffusion or surface treatment [50]. They related the water-oxidation surface states to the hematite (104) plane. High temperature annealing causes lattice distortion and deformation-direct Sn-doping, decreasing the density of favored surface states and causing an anodic shift in photocurrent onset potential. The performance of Sn-doped hematite depends collectively on surface states and donor density.

Pt is also an effective element that boosts hematite photoanode activity. Homogeneous distribution of Pt (3%–7%) in α-Fe_2_O_3_ was successfully prepared by simple electrodeposition route [55]. The Pt-doped nanoparticles were more densely packed and detailed structure characterization by Raman, XPS and XRD confirmed the incorporation of Pt^4+^ in the hematite lattice. Due to the increased donor concentration, the photocurrent increased from 0.69 mA/cm^2^ to 1.43 mA/cm^2^ at 0.4 V vs. Ag/AgCl. The doping-facilitated charge separation was also supported by a linear dependency of photocurrent on light intensities. Inspired by this work, Kim et al. employed Pt as dopant in worm-like hematite nanorods. The increased donor density and charge separation in Pt-Fe_2_O_3_ were confirmed by Mott-Schottky and EIS measurements. Moreover, the drop in charge transfer resistance at the interface suggested that Pt doping substantially accelerated the hole transfer to water and the catalytic oxygen evolution. Surface modification with Co-Pi catalyst gave a record-breaking photocurrent density of 4.32 mA/cm^2^ under standard conditions [56]. The remarkable feature of Pt doping was recently investigated by DFT + *U* calculations [54]. It was shown that Pt donates electrons to Fe sites, increasing the polaron hopping probability, and the electrical conductivity, but Pt substitution at the surface increases the free energy for O-H cleavage in water oxidation intermediates and decreases water adsorption at catalytic sites, which leads to high overpotential. Based on the calculation results, a gradient doping strategy has been proposed with low doping levels near the surface region to avoid the adverse effect.

### 2.2. p-Type Doping

**Figure 5:**
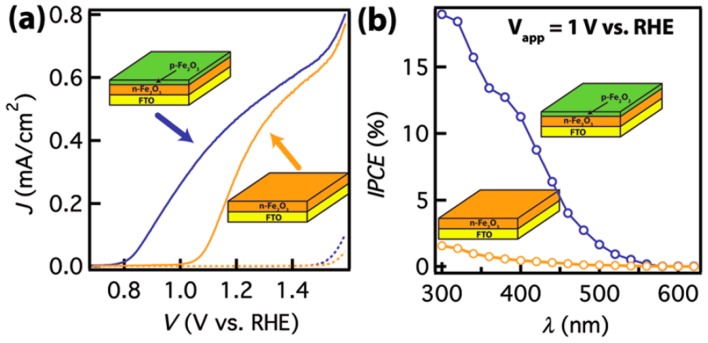
Photoelectrochemical characterizations of n-type Fe_2_O_3_ with and without p-type coating (total thickness: 25 nm). (**a**) A significant reduction of the turn-on voltage was observed on the sample with p-Fe_2_O_3_. Data measured under 1 Sun conditions (100 mW/cm^2^, AM 1.5 G). Dark currents shown in dashed lines; (**b**) IPCE characteristics of these samples at 1 V vs. RHE. Adapted with permission from [57]. Copyright 2012 American Chemical Society.

Mg [57,58] and Zn [59,60,61,62,63] are broadly exploited for p-type doping. In contrast to the metal elements addressed above, they create new states above the hematite valance band. High doping density gives p-type hematite, which is a suitable candidate as an overlayer to hinder charge recombination by forming p-n junctions at the surface, offering an additional charge separation driving force from an increased build-in field potential. In 2012, Lin et al. [57] constructed a first high quality n-p junction by growing Mg-doped hematite overlayer on top of n-type hematite. As shown in Figure 5, compared to the control photoanode, hematite with the p-type overlayer shows a ~200 mV cathodic shift in onset potential and much higher IPCE value at 1 V vs. RHE. The built-in field-assisted charge separation was verified by interfacial hole transfer kinetics extracted from EIS and open circuit potential measurements [57]. Hou et al. prepared a p-n heterojunction hematite photoanode with a Mg-doped overlayer (MgFe_2_O_4_) featuring favorable charge transfer properties. The 3-dimension branched architecture facilitates surface water oxidation, leading to a benchmark photocurrent density of 3.34 mA/cm^2^ at 0.8 V vs. Ag/AgCl [58].

**Figure 6:**
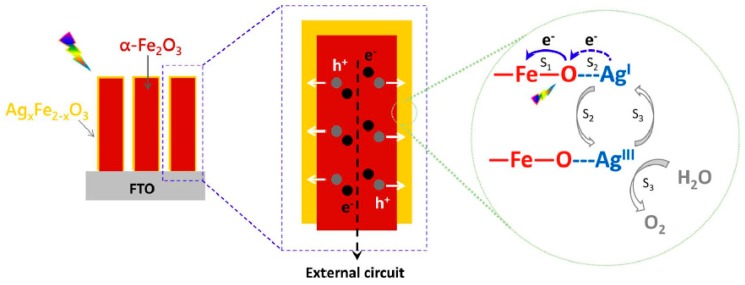
Core/shell structured α-Fe_2_O_3_/Ag_x_Fe_2-x_O_3_ nanorods and schematic of water oxidation reaction accelerated by Ag_x_Fe_2−x_O_3_ overlayer. Adapted with permission from [64]. Copyright 2014 Nature Publishing Group.

Successful PEC enhancement by p-type overlayer has also been reported for other metal dopants. Shen et al. used ultrasonication treatment of solution-based FeOOH in Ag acetate solution and obtained a Ag-doped overlayer on the hematite nanorods (α-Fe_2_O_3_/Ag_x_Fe_2−2x_O_3_ core shell structure). A 340% improved PEC water oxidation activity was observed in the optimized structure, in comparison to pristine hematite. Extensive Fe(3d)-O(2p) mixing in the valence band and greater contribution from O_2p_ holes was confirmed by in-situ X-ray absorption near edge structure (XANES) characterization. Concomitantly, a charge transfer between Ag^I^ and photogenerated hole gives Ag^III^ that promotes water oxidation (Figure 6) [64].

Zn doping was reported to greatly reduce the onset potential particularly by its contribution to the surface states and the faster surface hole transfer kinetics. In an effort to combine the better charge transport property by n-doping with the catalytic effect of Zn doping, Ti/Zn co-doped hematite was prepared by electrodeposition method with minimal change in morphology/optical properties[53]. Superior overall performance was observed compared to Ti-doped photoanode. Characterization by EIS suggested that Zn co-doping was beneficial for both charge transport in the bulk and water oxidation at the surface, the latter was supported by a two-fold increase in Tafel slope. This work, and other related studies pave the way to collaborate different mechanisms to promote solar water oxidation at doped hematite photoanodes.

## 3. Nonmetal Element Doping

**Figure 7:**
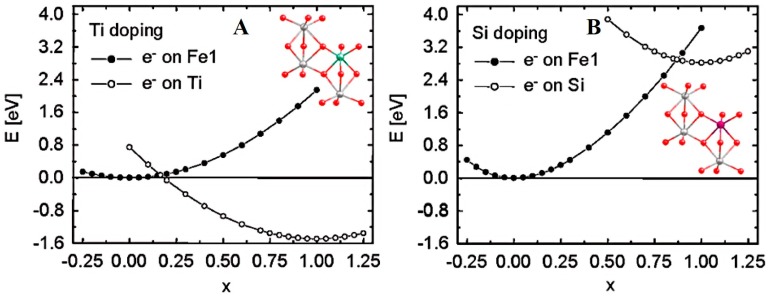
Unrestricted Hartree-Fock (UHF) potential energy curves along the nuclear reaction coordinates for electron transfer between Fe1 and (**A**) Ti with Ti doping (**B**) Si with Si doping. The hollow and solid circles represent actual calculation data points. The energy for x = 0 on the left curve is set to zero. Adapted with permission from [82]. Copyright 2011 American Chemical Society.

The nonmetal dopants for hematite have been limited to Si [31,67,68,69,70] and P [33,34] so far. Ab initio quantum mechanics calculations on the carrier transport property of doped hematite suggested that the nonmetallic Si could be superior to Ti [82]. As shown in Figure 7A, in Ti-doped hematite, the potential energy curve reveals that electron carrier located at Ti sites is ~1.5 eV more stable than that at Fe sites. Consequently, photogenerated electron carriers prefer to reside at Ti site, i.e., Ti dopant acts as electron trapping site and hinders electron transport. While for the Si-doping, the electron carrier strongly prefers Fe site owing to a ~2.8 eV gain in potential energy (Figure 7B). The difference in electron transfer trend was rationalized by electron accepting orbitals: Ti^4+^ has empty 3d orbital favoring electron trapping, which may explain the absence of Fe^2+^ in Ti-doped hematite [43]. For Si, on the contrary, electron is forced to occupy the empty Si-O antibonding orbitals, which is a highly unfavored process. Thereby, the nonmetal Si dopant offers electron carriers without affecting the original polaron hopping mode for electron transport along iron atoms, which is more advantageous to improve the hematite conductivity and PEC water oxidation activity. The generation of electron polaron by electron transfer from Si to surrounding iron site was reproduced by DFT + *U* calculations, elucidating the origin for electrical conductivity enhancement [83].

### 3.1. Si-Doping

**Figure 8:**
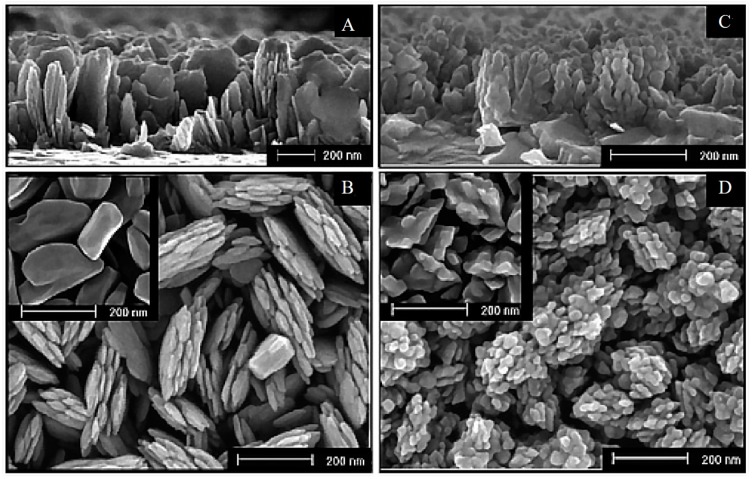
Typical HR-SEM images of Si-doped hematite films on TCO obtained from USP (**A**,**B**) and APCVD (**C**,**D**): a and c are side-view, b and d top-view images; (**B**, Inset) Hematite grains for undoped USP (**D**, Inset) Hematite grains for undoped APCVD electrodes. Adapted with permission from [67]. Copyright 2006 American Chemical Society.

Si-doping has been widely studied in the past decade [31,67,68,69,70]. In 2006, Cesar et al. reported the preparation of Si-doped hematite by ultrasonic spray pyrolysis (USP) and atmospheric pressure chemical vapor deposition (APCVD) with tetraethoxysilane (TEOS) as silicon dopant [67]. Improvement of 50% and 90% in photocurrent densities was realized for the Si-doped photoanodes prepared by the two protocols. The high PEC performance was ascribed to the decreased feature size of the nanoleaflets (Figure 8), as well as the improved conductivity resulting from Si-doping. This study highlights the importance of Si in developing the favored nanostructure for hole transfer, an effective solution to the short hole diffusion length of hematite.

**Figure 9:**
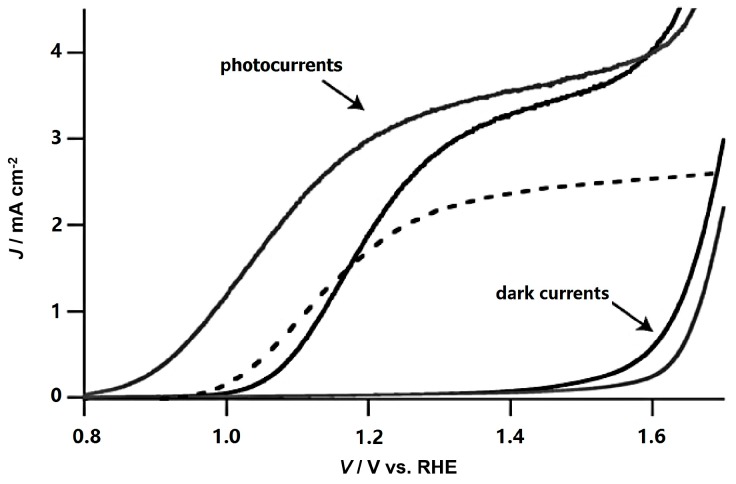
Performance of the unmodified hematite photoanode (solid black trace), and the same anode that was functionalized with IrO_2_ nanoparticles (solid gray trace). Conditions: 1 M NaOH solution (pH 13.6), 10 mV/s scan rate, uncorrected for Ohmic losses. The corresponding dark currents are also shown. The dashed trace is the photocurrent for the former state-of-the-art hematite photoanode. Adapted with permission from [31]. Copyright 2010 John Wiley & Sons.

Following this pioneering work, Kay et al. fabricated a dendritic Si-doped hematite photoanode by APCVD which gave a benchmark photocurrent of 2.2 mA/cm^2^ at 1.23 V vs. RHE. Such high efficiency was attributed to the nanostructure size features, minimizing the traveling distance of photogenerated holes [69]. Further enhancement was achieved by an ultrathin insulating SiO_2_ layer on the FTO substrate (to block back electron hole recombination) and the decoration of a catalytic cobalt layer. Properties including crystallinity disorder, feature sizes and donor carrier density of hematite by APCVD under various operating conditions was investigated systematically, identifying a critical feature sizes for the active photoanode. The Si introduced unusually high donor density was confirmed to be essential for the formation of space charge field and the enhanced water oxidation efficiency. With these mechanistic insights, Tilley et al. obtained the benchmark activity of 3.3 mA/cm^2^ at 1.23 V vs. RHE with an IPCE value of 39% at 400 nm in the presence of iridium oxide catalyst overlayer (Figure 9) [31]. Considering its atomic size, Si^4+^ was also employed as co-dopant to balance the radius difference in Ti-Fe_2_O_3_ (Si^4+^ < Fe^3+^ < Ti^4+^). The co-doped sample shows more than a two-fold increase in donor density and photocurrent in comparison to Ti-doped hematite [71].

### 3.2. P-Doping

**Figure 10:**
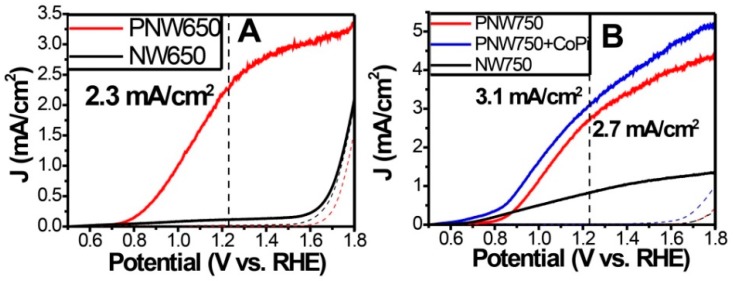
PEC performance of P-doped hematite photoanode. (**A**) *J-V* curves for the P-doped hematite anodes annealed at 650 °C under simulated AM 1.5 G illumination (solid lines) and in the dark (dash lines) in 1 M NaOH at a scan rate of 50 mV/s; (**B**) *J-V* curves for the P-doped hematite anodes annealed at 750 °C and “Co-Pi”-loaded P-doped hematite anode. Adapted with permission from [33]. Copyright 2015 Royal Society of Chemistry.

Recently our group introduced P-doped hematite to the photoanode catalogue for efficient solar water splitting [33,34]. Considering that phosphorous has five valence electrons (one more than Si) and the covalent nature of P-O bonds, P-doping was expected to be an effective strategy to avoid formation of electron trapping states. During the hydrothermal preparation of hematite electrode, we developed an impregnation process for P-doping [33]. The scanning transmission electron microscope (STEM) images and XPS depth profile confirmed the homogeneous distribution of P in bulk hematite. At optimal impregnation pH, the P-doped hematite photoanodes exhibited pronounced photocurrent densities of 2.3 mA/cm^2^ and 2.7 mA/cm^2^ at 1.23 V vs. RHE (annealed at 650 °C and 750 °C, denoted by PNW650 and PNW750) as shown in Figure 10. Loading of “Co-Pi” catalyst on the surface of PNW750 further increased the photocurrent density to 3.1 mA/cm^2^ at 1.23 V vs. RHE. DFT + *U* calculation revealed the greatly reduced electron effective mass upon P-doping, which is responsible to the substantial increase in electron mobility and the PEC activity.

**Figure 11:**
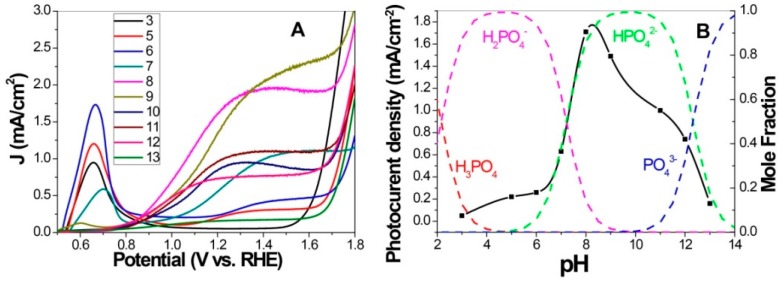
Effect of soaking solution pHs on hematite performance. (**A**) *J-V* scans collected for P-doped hematite synthesized by phosphate solutions with different pH and annealed at 650 °C. (**B**) Photocurrent densities obtained at 1.23 V_RHE_ and molar fraction of H_x_PO_4_^(3−x)−^ species as a function of pH. Adapted with permission from [33]. Copyright 2015 Royal Society of Chemistry.

During the preparation of the photoanode, it was found that solution pH in the impregnation process greatly affected the PEC performance of P-doped photoanodes. A systematical investigation indicated that high photocurrent density was obtained only when the soaking pH was around 8–9, as shown in Figure 11. Interestingly, comparison of Fe K-edge XANES spectra and P K-edge XANES spectra suggested an opposite trend in valence state between Fe and P. This provides the evidence for the doping introduced change in Fe valence state. As discussed above, extra electrons donated by doped element are critical for electron transport and PEC activity [84]. On the other hand, photoanode treated in acidic solution lost the well-defined crystallinity along (110) direction, which is detrimental to the activity based on the highly anisotropic electron mobility favoring this direction [2,46]. Overall, although acid treatment gives more Fe (II), the poor crystallinity hinders charge transfer, resulting in the observed low activity. From the above results, the novel P-doped hematite not only exhibited high PEC activity, but also provided a model system for studying the influences of iron valence state and crystallinity on the PEC behavior. Fine tune of these two parameters by a simple solution method is unprecedented in other doping studies.

## 4. Summary and Outlook

Although elemental doping has been intensely studied over the past years, the PEC activities of the doped hematite photoanodes via different synthetic routes are still far below the theoretical limitations of this material. There are important issues to be further explored in detail.

The specified efficiency-enhancement mechanisms and the major role played by dopants should be clarified. Most significantly, elements with extra valence electrons, represented by Ti, Sn, Pt, Si and P, increase the charge collection efficiencies by introducing additional electron carriers and/or tuning space charge layer; recent theoretical and experimental researches suggested that Si and P doping is more advantageous in terms of electron mobility and charge transport. The heteroatoms have the potential to promote the surface hole transfer (water oxidation) by changing the near surface electronic structure. However, the chemical nature and activity of the surface states involved in water oxidation remain elusive. From this point of view, bulk-sensitive and surface-sensitive characterizations, as well as theoretical modelling are necessary to identify the prevalent mechanisms. Whether these dopants can extend the carrier lifetime (fs-ps timescale) under cell operation is yet to be addressed. These insights are vital to guide the design of photoanodes by taking advantages of different dopant (co-doping) and control their spatial distribution to maximize the beneficial effects.

It has to be noted that the underlying mechanism and role of dopants depend on how electrode fabrication methods control the dopant “activation” process or their incorporation mode. Dopant alternated feature size and morphology also need to be addressed. Therefore, it is essential to explore these synergies for optimal performance.

Emerging new strategies, including high surface area 3D conducting substrate scaffold, metal nanostructure introduced buried Schottky junction, and plasmonic effect have shown promises in improving hematite performance. These methods, in combination with elemental doping, are envisioned to operative collectively to achieve high solar to fuel conversion efficiency.
